# The association of payer type and opioid use on functional improvement at short-term follow-up after lumbosacral transforaminal epidural steroid injection: Results of a large registry study

**DOI:** 10.1016/j.inpm.2022.100073

**Published:** 2022-02-17

**Authors:** Raquel Reisinger, Marc Caragea, Masaru Teramoto, Hank Shipman, Dennis Berry-Rieser, Muna Oli, Richard Kendall, Taylor Burnham, Aaron Conger, Zachary McCormick

**Affiliations:** a Department of Human Genetics, Utah Center for Genetic Discovery, University of Utah, Salt Lake City, UT, USA; b School of Medicine, University of Utah, Salt Lake City, UT, USA; c Department of Physical Medicine and Rehabilitation, University of Utah Health, Salt Lake City, UT, USA

**Keywords:** ESI, MCID, ODI, Insurance, Spine, Pain medicine, Low back pain, Radicular pain

## Abstract

**Background:**

Lumbosacral transforaminal epidural steroid injection (LTFESI) is a commonly performed intervention for treating radicular pain. While factors that predict pain improvement after LTFESI have been evaluated, minimal literature exists regarding predictors of functional improvement.

**Purpose:**

To identify factors that are associated with functional improvement at short-term follow-up after LTFESI.

**Study design:**

Retrospective review of prospectively collected registry data.

**Patient sample:**

Patients undergoing LTFESI at an academic spine center who completed an Oswestry Disability Index (ODI) questionnaire both pre-procedure and one to three weeks post-procedure.

**Outcome measures:**

The outcomes of interest were the proportions of patients who experienced a minimal clinically important difference (MCID) in function defined as ​≥ ​30% improvement in ODI score, as well as ​≥ ​10-point and ≥15-point improvement in ODI score.

**Methods:**

Logistic regression analysis was performed to examine the associations of predictor variables to the ODI responder/non-responder outcome variable. The predictor variables for the analysis included: age, baseline ODI score, Charleston Comorbidity Index (CCI), payer type, prior lumbosacral spine surgery, pre-injection opioid use, two-level injections, bilateral injections, repeat injection, trainee presence during injection, immediate numerical rating scale (NRS) change post-injection. An odds ratio (OR) and its 95% confidence intervals (CIs) were calculated.

**Results:**

A total of 606 patients were included in the analysis. More than half of the patients (56.8%) reported a ≥7.1% improvement in ODI score, and about 30% reported a ≥30% improvement in ODI score. Approximately 36% and 20% of the patients reported ≥10-point and ≥15-point reductions in ODI score, respectively. Medicaid and Medicare payer type and pre-injection opioid use were significantly associated with a lower likelihood of ≥30%, and ≥15-point improvements in ODI, after adjusting for the other factors (*p* ​< ​0.05).

**Conclusions:**

When using various common definitions of MCID for ODI score improvement, Medicaid, Medicare, and pre-injection opioid use were identified as factors that are negatively associated with functional improvement at short-term follow-up after LTFESIs.

## 1 Introduction

Low back pain (LBP) is a common and costly problem in the United States, with approximately 80% of the population experiencing LBP at some time in their life [1,2]. It is the leading cause of work-related disability in individuals under 45, and costs related to LBP exceed $100 billion per year in the United States [3]. According to the 2010 World Health Organization Global Burden of Disease Report, LBP is the leading cause of disability and 6th in overall burden [4].

Lumbosacral radicular pain is often associated with LBP and is often associated with disability when compared to axial LBP alone [5,6]. First-line treatments of lumbosacral radicular pain include non-invasive therapies such as activity modification, analgesic medications, and formal physical therapy with a personalized home exercise program [7]. While opioids are sometimes prescribed, their long-term use is not recommended, given high rates of associated dependence, addiction, and adverse events [8].

Lumbosacral transforaminal epidural steroid injections (LTFESIs) are an effective treatment option to reduce pain in patients who have little to no improvement with more conservative management [9,10]. Previous studies have identified factors associated with treatment success following a LTFESI for lumbosacral radicular pain [[11], [12], [13], [14]]. Disc herniation, shorter duration of symptoms, positive neural tension signs on exam, and imaging consistent with mild nerve root compression have been associated with a greater likelihood of pain reduction following a LTFESI [[11], [12], [13], [14]]. While factors that predict pain improvement have been evaluated, there is minimal literature regarding factors that predict functional improvement following LTFESIs.

In 2016, Sivaganesan et al. [15] investigated predictors of functional improvement after lumbar epidural steroid injections. However, the authors defined meaningful functional improvement as a 7.1% decrease on the Oswestry Disability Index (ODI), a substantially smaller change than the minimal clinically important difference (MCID) of 10-point, 15-point, or 30% improvements in ODI, as commonly defined in the LBP literature [[16], [17], [18], [19]]. Therefore, there is a need to establish predictors of ODI improvement based on common and more robust MCID definitions.

As such, the present study aimed to determine factors that predict functional improvement following LTFESI at short-term follow-up using robust, clinically meaningful improvement in function as measured by the ODI in a large cohort of patients.

## 2 Methods

### 2.1 Data collection

This single-center retrospective review of prospectively collected registry data was approved by the University of Utah Institutional Review Board (IRB 00069703). The electronic medical records of consecutive patients who underwent LTFESI from November 2013 to May 2018 were reviewed after first being identified through electronic database search of CPT codes for LTFESI (64483). All records were reviewed for patients who met the pre-determined inclusion criteria: 18–89 years old who underwent a LTFESI and completed the ODI questionnaire both pre-procedure and one to three weeks post-procedure. The exclusion criteria were: <18 years or >89 years old and incomplete ODI questionnaire or baseline ODI score of 0. Interlaminar epidural steroid injections and transforaminal steroid injections performed in the cervical or thoracic spine were excluded. Transforaminal injections using anesthetic alone without steroid were also excluded. The predictor variables for the analysis included: age, baseline ODI score, Charleston Comorbidity Index (CCI) score, payer type, prior lumbosacral spine surgery, pre-injection opioid use, two-level injections, bilateral injections, repeat injection, trainee presence during injection, immediate numerical rating scale (NRS) change post-injection. Payer type was determined based on the primary coverage listed in the patient’s electronic medical record. Payer types assessed included Medicare, Medicaid, private, government, and workers compensation. Government insurance constituted of non-Medicare/Medicaid government healthcare programs (TRICARE, Veterans Health Administration, Post Employment Health Plan). The study was conducted according to the Declaration of Helsinki.

### 2.2 Procedures

All LTFESI injections were performed by physicians specializing in Physical Medicine and Rehabilitation with fellowship training in either Pain Medicine or Sports Medicine.

### 2.3 LTFESI technique

All LTFESIs were performed using a subpedicular or infraneural transforaminal approach as outlined by the Spine Intervention Society guidelines [20]. Patients were positioned on a table in the prone position, and a time-out procedure was performed. The appropriate level was confirmed under fluoroscopy. The lumbosacral region was prepped with an iodine solution x3 or chlorhexidine and draped in a sterile fashion. The skin and subcutaneous tissues were then anesthetized with 1–2 ​mLs of 1% lidocaine. Under direct fluoroscopic guidance, a 22 or 25-gauge 3.5-, 5-, or 7-inch spinal needle was directed obliquely to enter the superior-most aspect of the neuroforamen for a subpedicular approach or the inferior, retrodiscal aspect of the neuroforamen for an infraneural approach. Needle position was confirmed in both AP and lateral views. Next, 1–2 ​cc of iodinated contrast medium or gadolinium-based contrast medium was injected under fluoroscopic observation, unless contraindicated due to allergy. The needle tip was repositioned as needed until contrast was observed to highlight the exiting nerve root producing adequate proximal flow into the epidural space without vascular uptake. The injection was then completed with 1 ​cc of dexamethasone (10 mg/cc) and 1.5 ​cc of 2% preservative-free lidocaine. Bilateral and multilevel injections were performed at the discretion of the treating physician based on patient symptoms. Patients with bilateral symptoms underwent bilateral injections, and patients with multi-level disease underwent 2-level injections. No patients underwent a procedure targeting more than two injection sites.

### 2.4 Outcome Measures

ODI scores were measured both pre-procedure and at one to three weeks post-LTFESI. The primary outcome was the proportion of responders that achieved various common robust definitions of the MCID of ≥10, ≥15 points, and ≥30% reduction on the ODI. The secondary outcome was the proportion of responders that achieved ≥7.1% improvement on ODI for comparison to previous study [15], but recognized to be a low threshold definition of “treatment success”.

### 2.5 Data analysis

Descriptive statistics were calculated for demographic, clinical, and procedural characteristics. Specifically, mean and standard deviation (SD) calculations were used for continuous variables, while categorical variables were summarized using frequencies and percentages. Logistic regression analysis was performed to examine the associations of predictor variables to the ODI-related, dichotomous outcome variables above. An odds ratio (OR) and its 95% confidence interval (CI) were calculated for each predictor variable in the logistic regression models. An *α* level was set at 0.05, and all the analyses were conducted using Stata 16.1 (StataCorp LLC, College Station, TX).

## 3 Results

A total of 724 patients were screened, 606 of which met the inclusion criteria. The mean age was 56.0 ​± ​15.9 years, and the mean baseline ODI score within the cohort was 41.4 ​± ​17.1. Approximately 33% percent of patients were insured under Medicare, 6% under Medicaid, 49% under private, 8% under government and 5% under workers compensation. Forty-six percent of the cohort had used opioids in the past month. Table 1 summarizes the demographics, clinical characteristics, and insurance types of the included patients.

**Table 1:** Demographics, clinical, and procedural characteristics (N ​= ​606).

Variable	mean (SD) or n (%)
Age in years	56.0 (15.9)
Baseline ODI score	41.4 (17.1)
CCI score	1.5 (2.4)
Payer type	
Medicare	201 (33.2)
Medicaid	36 (5.9)
Private	294 (48.5)
Government	47 (7.8)
Workers	28 (4.6)
Prior lumbosacral spine surgery	
Yes	31 (5.1)
No	575 (94.9)
Pre-injection opioid use	
Yes	279 (46.0)
No	327 (54.0)
Two-level injections	
Yes	167 (27.6)
No	439 (72.4)
Bilateral injections	
Yes	126 (20.8)
No	480 (79.2)
Repeat injection	
Yes	135 (22.3)
No	471 (77.7)
Trainee present during injection	
Yes	196 (32.3)
No	408 (67.3)
Immediate NRS change post-injection	3.8 (2.6)

Overall, approximately 36% and 20% of patients achieved ≥10-point and ≥15-point improvements in ODI, respectively. Twenty-nine percent of patients reported a ≥30% improvement in ODI score after LTFESI. More than half of patients (56.8%) reported a ≥7.1% improvements in ODI score. The responder analysis based on these four different thresholds is summarized in Table 2.

**Table 2:** Responder analysis according to various ODI score improvement thresholds.

Variable	Yes	No	95% CI for Yes
≥10-point reductions in ODI	220 (36.3)	386 (63.7)	32.6–40.2
≥15-point reductions in ODI	119 (19.6)	487 (80.4)	16.7–23.0
≥30% reductions in ODI	177 (29.2)	429 (70.8)	25.7–33.0
≥7.1% reductions in ODI	345 (56.9)	261 (43.1)	52.9–60.8

Values are counts (% frequency).

CI ​= ​confidence interval.

The results of the logistic regression analysis are presented in Table 3. Payer type was significantly associated with a likelihood of attaining ≥15-point improvement in ODI score following TFESI, after adjusting for the other predictors (*p* ​< ​0.05). Specifically, compared with patients covered by private insurance, patients with Medicare were more than two times less likely to report ≥15-point improvement in ODI score, and patients with Medicaid were more than four times less likely to report this magnitude of functional improvement (*p* ​= ​0.023; OR ​= ​0.48, 95% CI ​= ​0.26–0.90 for Medicare and *p* ​= ​0.010; OR ​= ​0.24, 95% CI ​= ​0.08–0.72 for Medicaid). When controlling for the other predictors, payer type was also significantly associated with the likelihood of achieving ≥30% improvement in ODI score (*p* ​< ​0.05). Using this responder threshold, patients with Medicare were nearly two times less likely to report ≥30% improvement in ODI score, and patients with Medicaid were more than three times less likely to report ≥30% improvement in ODI score after LTFESI (*p* ​= ​0.049; OR ​= ​0.59; 95% CI ​= ​0.35–0.99 for Medicare and *p* ​= ​0.027; OR ​= ​0.29; 95% CI ​= ​0.10–0.87 for Medicaid).

**Table 3:** Logistic regression models on ODI predictive variable.

Outcome	Predictor		OR	95% CI	*p*
≥10-point reductions in ODI	Age		1.01	0.99–1.02	0.208
	Baseline ODI score		1.04	1.02–1.05	<**0.001**
	CCI score		0.99	0.92–1.07	0.842
	Payer type (vs. private)	Medicare	0.62	0.37–1.02	0.062
		Medicaid	0.56	0.25–1.24	0.154
		Government	0.75	0.37–1.54	0.436
		Workers	0.60	0.25–1.43	0.249
	Prior lumbosacral spine surgery (vs. surgically naïve)	Yes	0.63	0.26–1.50	0.295
	Pre-injection opioid use (vs. no)	Yes	0.62	0.43–0.90	**0.011**
	Two-level injections (vs. single level)	Yes	0.45	0.29–0.70	<**0.001**
	Bilateral injections (vs. unilateral)	Yes	0.99	0.64–1.53	0.957
	Repeat injection (vs. first injection)	Yes	1.18	0.76–1.82	0.461
	Trainee present during injection (vs. no trainee)	Yes	0.58	0.40–0.86	**0.006**
	Immediate NRS change post-injection		0.98	0.91–1.05	0.538
≥15-point reductions in ODI	Age		1.01	0.99–1.03	0.299
	Baseline ODI score		1.05	1.04–1.07	<**0.001**
	CCI score		1.01	0.91–1.11	0.882
	Payer type (vs. private)	Medicare	0.48	0.26–0.90	**0.023**
		Medicaid	0.24	0.08–0.72	**0.010**
		Government	0.52	0.20–1.36	0.183
		Workers	0.58	0.20–1.69	0.321
	Prior lumbosacral spine surgery (vs. surgically naïve)	Yes	0.45	0.13–1.62	0.224
	Pre-injection opioid use (vs. no)	Yes	0.46	0.29–0.73	**0.001**
	Two-level injections (vs. single level)	Yes	0.81	0.48–1.38	0.441
	Bilateral injections (vs. unilateral)	Yes	0.94	0.54–1.62	0.816
	Repeat injection (vs. first injection)	Yes	0.93	0.54–1.61	0.792
	Trainee present during injection (vs. no trainee)	Yes	0.64	0.40–1.04	0.073
	Immediate NRS change post-injection		1.00	0.92–1.09	0.981
≥30% reductions in ODI	Age		1.00	0.99–1.02	0.711
	Baseline ODI score		0.99	0.98–1.01	0.381
	CCI score		0.95	0.86–1.04	0.241
	Payer type (vs. private)	Medicare	0.59	0.35–0.99	**0.049**
		Medicaid	0.29	0.10–0.87	**0.027**
		Government	0.51	0.24–1.06	0.072
		Workers	0.53	0.21–1.32	0.174
	Prior lumbosacral spine surgery (vs. surgically naïve)	Yes	0.66	0.27–1.63	0.365
	Pre-injection opioid use (vs. no)	Yes	0.57	0.39–0.83	**0.003**
	Two-level injections (vs. single level)	Yes	0.73	0.47–1.13	0.163
	Bilateral injections (vs. unilateral)	Yes	0.82	0.52–1.31	0.408
	Repeat injection (vs. first injection)	Yes	1.24	0.79–1.93	0.344
	Trainee present during injection (vs. no trainee)	Yes	0.63	0.42–0.94	**0.023**
	Immediate NRS change post-injection		0.96	0.89–1.04	0.340
≥7.1% reductions in ODI	Age		1.01	1.00–1.03	0.092
	Baseline ODI score		1.01	1.00–1.02	0.104
	CCI score		0.94	0.87–1.01	0.081
	Payer type (vs. private)	Medicare	0.80	0.50–1.29	0.367
		Medicaid	0.66	0.32–1.39	0.272
		Government	0.79	0.42–1.50	0.471
		Workers	1.23	0.54–2.82	0.624
	Prior lumbosacral spine surgery (vs. surgically naïve)	Yes	0.67	0.31–1.42	0.294
	Pre-injection opioid use (vs. no)	Yes	0.64	0.45–0.89	**0.009**
	Two-level injections (vs. single level)	Yes	0.60	0.41–0.88	**0.009**
	Bilateral injections (vs. unilateral)	Yes	1.05	0.69–1.60	0.825
	Repeat injection (vs. first injection)	Yes	1.11	0.74–1.67	0.614
	Trainee present during injection (vs. no trainee)	Yes	0.80	0.56–1.13	0.208
	Immediate NRS change post-injection		0.99	0.93–1.06	0.813

OR ​= ​odds ratio; CI ​= ​confidence interval.

Pre-injection opioid use was significantly associated with ≥10-point, ≥ 15-point, ≥ 30%, and ≥7.1% improvements in ODI score (*p* ​< ​0.05), when controlling for the other predictors. Patients using opioids were nearly one and a half times less likely to report ≥10-point and ≥7.1% improvements in ODI score, and approximately two times less likely to report ≥15-point and ≥30% improvements in ODI score following TFESI (*p* ​= ​0.011; OR ​= ​0.62; 95% CI ​= ​0.43–0.90 for ≥10-point, *p* ​= ​0.009; OR ​= ​0.64; 95% CI ​= ​0.45–0.89; for ≥7.1%, *p* ​= ​0.003; OR ​= ​0.57; 95% CI ​= ​0.39–0.83 for ≥30%, and *p* ​= ​0.001; OR ​= ​0.46; 95% CI ​= ​0.29–0.73 for ≥15-point).

Two-level injections were significantly associated with the likelihood of ≥10-point and ≥7.1% improvements in ODI score, after adjusting for the other predictors (*p* ​< ​0.05). When compared with patients receiving a single injection, those with two-level injections were over two times less likely to report ≥10-point improvement in ODI and more than one and a half times less likely to report ≥7.1% reduction in ODI score (*p* = ​< ​0.001; OR ​= ​0.45; 95% CI ​= ​0.29–0.70 for ≥10-point and *p* ​= ​0.009; OR ​= ​0.60; 95% CI ​= ​0.41–0.88 for ≥7.1%).

Trainee presence during injection was also significantly associated with ≥10-point and ≥30% reductions in ODI, after adjusting for the other predictors (*p* ​< ​0.05). Specifically, when compared with no trainee, patients with a trainee present during the LTFESI were over one and a half less likely to report both ≥10-point and ≥30% reductions in ODI (*p* ​= ​0.006; OR ​= ​0.58; 95% CI ​= ​0.40–0.86, for ≥10-point, and *p* ​= ​0.023; OR ​= ​0.63; 95% CI ​= ​0.42–0.94 for ≥30%)

## 4 Discussion

Sivaganesan et al. [15] reported 62% of patients achieved a 7.1% improvement in ODI following lumbar epidural steroid injections. In comparison with that study, we report similar findings of 56.8% of patients meeting a 7.1% reduction threshold in ODI following LTFESI. However, we recognize this threshold to be too low to be considered a clinically meaningful important difference in ODI. This study established predictors of ODI score reductions based on more stringent criteria for MCID thresholds more congruent with ODI achievements commonly defined in the LBP literature. Additionally, unlike prior studies which have primarily assessed pain reduction, this study investigated factors associated with short-term clinically meaningful functional improvement following LTFESI in a large single-center cohort from registry data. As the number of LTFESIs performed in the United States increases annually [21], physicians must have a better model to evaluate candidates who are likely to experience both pain and functional improvement post-injection. The results of this study can be used to aid in the identification of predictors for treatment success. Using these predictors, providers can gain valuable insight into the identification of patients who have a greater likelihood of gaining clinically meaningful functional improvement at short-term follow-up after lumbosacral TFESIs. Furthermore, the potential for modification of these predictors for treatment success provides a compelling opportunity for improving patient care and reducing the economic and financial burden of LBP in the United States.

This study found that compared with patients covered by private insurance, the odds of achieving ≥30%, and ≥15-point reductions in ODI were significantly lower for patients with Medicare or Medicaid. We controlled for various predictors such as age, comorbidities, and baseline ODI, neither of which were associated with an ODI score reduction. We hypothesize that the discrepancy in functional outcomes among payer types is likely a result of hidden confounders in socioeconomic and demographic disparities. Studies evaluating the outcome of mortality from major surgical operations, cardiac events after percutaneous coronary interventions, survival of patients with head and neck cancer, and long-term survival after lung transplantation similarly found worse outcomes and/or increased mortality in patients with Medicare or Medicaid compared with private insurance [[22], [23], [24], [25]]. This patient population is likely to have multiple barriers to receiving health care, including financial stressors limiting time off work, lack of transportation, and limited access to co-treatment interventions such as physical therapy [26]. Socioeconomic factors are inherently multidimensional, and may be prone to misinterpretation, highlighting the importance of further investigation and the development of reliable measures [27].

In the present study, the odds of achieving ≥7.1%, ≥30%, ≥10-point, and ≥15-point reductions in ODI for patients using opioids were significantly lower than the odds for non-opioid users. This is an additional novel finding, as prior studies have not identified opioid use to be significantly associated with functional response to epidural steroid injections in patients with radicular pain [15,28]. Similar findings have been reported in those undergoing spine surgery. Studies show that prolonged preoperative opioid utilization is associated with poor return to work rates and adverse patient reported outcomes after spinal surgery [29,30]. Despite numerous health risks, opioids remain a commonly prescribed treatment for low back pain despite the lack of strong evidence supporting their long-term efficacy [31]. When considering that almost half of our patient population had reportedly taken opioids within the past month pre-injection (46%), this presents a significant opportunity for potentially improving functional outcomes and overall patient care.

A two-level injection was significantly associated with worse functional outcomes in achieving the ≥7.1%, and ≥10-point reductions in ODI thresholds, but not observed in the ≥30% and ≥15-point thresholds. This may be due to more severe pathology that leads to the decision to perform a multi-level injection. History of spine surgery or repeat/bilateral injection was not associated with ODI improvement.

Finally, trainee presence was associated with a lower likelihood of achieving ≥30% and ≥10-point reductions on ODI, but not observed at the higher ≥15-point threshold. Interestingly, a large single-institution retrospective review also performed at the University of Utah assessed trainee involvement on fluoroscopic time, pain reduction, and complication rate in over 7,800 fluoroscopy-guided injections and found no significant difference in outcomes with trainee involvement [32]. Patient safety and functional outcomes are likely not compromised with appropriate trainee supervision, but further investigation is warranted.

As the population covered by Medicaid and Medicare continues to increase, payer status and socioeconomic factors have become a central focus of public health and health care reform initiatives [33]. Furthermore, with healthcare costs of low back pain exceeding $100 billion per year in the United States, knowledge of optimal patient selection for effective therapies has the potential for larger economic impacts [4]. Studies identifying predictive factors of functional response, specifically those including payer status and socioeconomic factors, are not well understood. The global scale of lumbosacral radicular pain and its evidenced effects on physical and mental health demonstrates the importance of this predictive modeling to identify patients who have a greater likelihood of gaining functional improvement after LTFESIs.

### 4.1 Study limitations and strengths

The primary limitations of this study include the lack of corresponding imaging data and information regarding duration of pain and functional impact to best identify diagnosis and etiology of symptoms. Patient specific diagnoses, such as central or neuroforaminal stenosis, disc herniation, or spondylolisthesis, was not available for analysis. Evaluating underlying pathology may help refine a model for predicting positive functional response. As a registry study, the results indicate association, but causation cannot be stated. Of note, outcome data was collected prospectively and entered into a registry in order to limit recall bias associated with a cross-sectional type study that may introduce recall bias. An additional limitation was assessment of only short-term outcomes. This was necessary as our registry contained high penetrance of follow-up outcomes at one-to-three-weeks post-injection, but loss-to-follow-up increased at intermediate to long-term time points post injection. Identifying additional clinical or procedural factors may have revealed other associations with functional improvement after LTFESI at a longer follow-up duration. Additionally, while our analysis controlled for comorbidities using the Charleston Comorbidity Index, we were unable to investigate the influence of mental health, race/ethnicity, education, or income from this specific registry dataset. Finally, only primary insurance coverage was included in the analysis. While the majority of patients have single coverage, adding secondary payer source information may have added additional insight for particular patients.

The available procedural factors and the large cohort size in this study provide an excellent opportunity to determine predictive factors of LTFESI response. We evaluated various common MCID thresholds to provide a detailed illustration of “treatment success” based on more rigorous definitions. Future studies should include imaging-based diagnosis to determine the influence on functional outcomes. Additionally, more investigation is necessary to better understand the factors underlying the impact of socioeconomic and demographic disparities in response to LTFESI. This study highlights the need for further investigation into the characteristics associated with treatment success after LTFESI.

## 5 Conclusion

We present the findings of a large cohort study of prospectively collected single-center registry data that provides a basis for aiding in identifying patients with a greater likelihood of clinically meaningful functional improvement after LTFESIs. When using various common definitions of MCID for ODI score improvement, Medicaid, Medicare, and pre-injection opioid use were negatively associated with functional improvement at short-term follow-up after LTFESIs. Further investigation on the influence of demographic and socioeconomic disparities on functional improvement is warranted.

## Declaration of competing interest

The authors declare the following financial interests/personal relationships which may be considered as potential competing interests:

Zachary L. McCormick, MD, serves on the Board of Directors of the Spine Intervention Society. There are no other potential conflicts of interest to disclose on the part of any of the other authors.

## References

[1] Friedly J., Standaert C., Chan L. (Nov. 2010).

[2] Deyo R.A., Weinstein J. (Feb. 2001). Low back pain. N Engl J Med.

[3] Crow W.T., Willis D.R. (2009). Estimating cost of care for patients with acute low back pain: a retrospective review of patient records. J Am Osteopath Assoc.

[4] Hoy D. (2014). The global burden of low back pain: estimates from the Global Burden of Disease 2010 study. Ann Rheum Dis.

[5] Singh J.R., Cardozo E., Christolias G.C. (Apr. 2017). The clinical efficacy for two-level transforaminal epidural steroid injections. Pharm Manag PM R.

[6] Koes B.W., van Tulder M.W., Peul W.C. (Jun. 2007). Diagnosis and treatment of sciatica. BMJ Br Med J.

[7] Macedo L.G. (Dec. 2013). Physical therapy interventions for degenerative lumbar spinal stenosis: a systematic review. Phys Ther.

[8] Martell B.A. (Jan. 16, 2007). Systematic review: opioid treatment for chronic back pain: prevalence, efficacy, and association with addiction. Ann Intern Med.

[9] Smith C.C., McCormick Z.L., Mattie R., MacVicar J., Duszynski B., Stojanovic M.P. (2020). The effectiveness of lumbar transforaminal injection of steroid for the treatment of radicular pain: a comprehensive review of the published data. Pain Med (United States).

[10] Ghahreman A., Ferch R., Bogduk N. (Aug. 2010). The efficacy of transforaminal injection of steroids for the treatment of lumbar radicular pain. Pain Med (United States).

[11] McCormick Z., Cushman D., Casey E., Garvan C., Kennedy D.J., Plastaras C. (Dec. 2014). Factors associated with pain reduction after transforaminal epidural steroid injection for lumbosacral radicular pain. Arch Phys Med Rehabil.

[12] McCormick Z.L. (Nov. 2016). Pain reduction and repeat injections after transforaminal epidural injection with particulate versus nonparticulate steroid for the treatment of chronic painful lumbosacral radiculopathy. Pharm Manag PM R.

[13] McCormick Z. (Dec. 2015). Comparison of pain score reduction using triamcinolone vs. Betamethasone in transforaminal epidural steroid injections for lumbosacral radicular pain. Am J Phys Med Rehabil.

[14] Ghahreman A., Bogduk N. (2011). Predictors of a favorable response to transforaminal injection of steroids in patients with lumbar radicular pain due to disc herniation. Pain Med.

[15] Sivaganesan A., Chotai S., Parker S.L., Asher A.L., McGirt M.J., Devin C.J. (Aug. 2016). Predictors of the efficacy of epidural steroid injections for structural lumbar degenerative pathology. Spine J.

[16] Kapural L. (2013). A randomized, placebo-controlled trial of transdiscal radiofrequency, biacuplasty for treatment of discogenic lower back pain. Pain Med (United States).

[17] Fischgrund J.S. (May 2018). Intraosseous basivertebral nerve ablation for the treatment of chronic low back pain: a prospective randomized double-blind sham-controlled multi-center study. Eur Spine J.

[18] Davidson M., Keating J.L. (2002). A comparison of five low back disability questionnaires: reliability and responsiveness. Phys Ther.

[19] Ostelo R.W.J.G. (Jan. 2008). Interpreting change scores for pain and functional status in low back pain: towards international consensus regarding minimal important change. Spine (Phila. Pa. 1976).

[20] Bogduk N. (2013).

[21] Manchikanti L., Pampati V., Falco F.J.E., Hirsch J.A. (2013). Assessment of the growth of epidural injections in the medicare population from 2000 to 2011. Pain Physician.

[22] Kwok J., Langevin S.M., Argiris A., Grandis J.R., Gooding W.E., Taioli E. (Jan. 2010). The impact of health insurance status on the survival of patients with head and neck cancer. Cancer.

[23] Lapar D.J. (Sep. 2010). Primary payer status affects mortality for major surgical operations. Ann Surg.

[24] Gaglia M.A. (Mar. 2011). Effect of insurance type on adverse cardiac events after percutaneous coronary intervention. Am J Cardiol.

[25] Allen J.G. (Jan. 2011). Insurance status is an independent predictor of long-term survival after lung transplantation in the United States. J Heart Lung Transplant.

[26] McCallum C.A. (2010). Access to physical therapy services among medically underserved adults: a mixed-method study. Phys Ther.

[27] Braveman P.A. (Dec. 2005). Socioeconomic status in health research: one size does not fit all. J Am Med Assoc.

[28] Wei J.J. (May 2018). Effect of pre-injection opioid use on post-injection patient-reported outcomes following epidural steroid injections for radicular pain. Spine J.

[29] Faour M. (2017). Prolonged preoperative opioid therapy associated with poor return to work rates after single-level cervical fusion for radiculopathy for patients receiving workers’ compensation benefits. Spine (Phila. Pa. 1976*)*.

[30] Lee D. (Jun. 2014). Preoperative opioid use as a predictor of adverse postoperative self-reported outcomes in patients undergoing spine surgery. J Bone Jt Surg - Am.

[31] Chaparro L.E., Furlan A.D., Deshpande A., Mailis-Gagnon A., Atlas S., Turk D.C. (Aug. 27, 2013).

[32] Cushman D., Teramoto M., Curtis B., Lee D.T., Marcolina A., McCormick Z. (2017). Does trainee involvement in fluoroscopic injections affect fluoroscopic time, immediate pain reduction, and complication rate?. Pharm Manag PM R.

[33] Bureau U.C. (2020). Poverty in the U.S.: trends, issues and measurements.

